# Colorectal Cancer: Molecular Mutations and Polymorphisms

**DOI:** 10.3389/fonc.2013.00114

**Published:** 2013-05-13

**Authors:** Aga Syed Sameer

**Affiliations:** ^1^Department of Biochemistry, Sher-I-Kashmir Institute of Medical Sciences Associated Medical College, Bemina, SrinagarKashmir, India

**Keywords:** colorectal cancer, mutations, hypermethylation, polymorphism, medical genetics

## Abstract

Colorectal cancer (CRC) is one of the major causes of mortality and morbidity, and is the third most common cancer in men and the second most common cancer in women worldwide. The incidence of CRC shows considerable variation among racially or ethnically defined populations in multiracial/ethnic countries. The tumorigenesis of CRC is either because of the chromosomal instability (CIN) or microsatellite instability (MIN) or involving various proto-oncogenes, tumor-suppressor genes, and also epigenetic changes in the DNA. In this review I have focused on the mutations and polymorphisms of various important genes of the CIN and MIN pathways which have been implicated in the development of CRC.

## Introduction

Colorectal cancer (CRC) defined as the cancerous growths in the colon, rectum and appendix is also referred to as colon cancer or large bowel cancer. It is a commonly diagnosed cancer in both men and women and represents the third most common form of cancer and the second leading cause of cancer-related death in the western world (Center et al., 2009). Sporadic CRC constitutes about 75% of the patients, with no apparent evidence of having inherited the disorder; while as the patients having a family history of CRC constitutes the remaining 25% which suggests a genetic contribution, common exposures among family members, or a combination of both (National cancer Institute, 2008^1^). In the countries like Western Europe, North America, and Australia CRC has the high incidence rates among the western world populations while as the lowest rates of CRC are found in the sub-Saharan Africa, South America and Asia, but are increasing in countries adopting western life-style and dietary habits (Vainio and Miller, 2003).

Colorectal tumors present with a broad spectrum of neoplasms, ranging from benign growths to invasive cancer, and are predominantly epithelial-derived tumors (i.e., adenomas or adenocarcinomas). Pathologists have classified the lesions into three groups: non-neoplastic polyps, neoplastic polyps (adenomatous polyps, adenomas), and cancers (Zauber et al., 2002; O’Brien et al., 2004).

### Types

Depending upon the genetics and the etiology of the disease, CRC is usually categorized into three specific types: sporadic, inherited, or familial.

#### Sporadic colorectal carcinomas

Sporadic carcinomas devoid of any familial or inherited predisposition, accounts for approximately 70% of CRC. Sporadic cancer is common in persons older than 50 years of age, probably as a result of dietary and environmental factors as well as normal aging. Fewer than 10% of patients have an inherited predisposition to colon cancer.

#### Inherited colorectal carcinomas

They include those in which colonic polyps are a major manifestation of disease and those in which they are not. The nonpolyposis predominant syndromes include hereditary nonpolyposis CRC (HNPCC) (Lynch syndrome I) and the cancer family syndrome (Lynch syndrome II). Although uncommon, these syndromes provide insight into the biology of all types of CRC.

#### Familial colorectal carcinomas

The third and least understood pattern of CRC development is known as familial CRC. In affected families, CRC develops too frequently to be considered sporadic but not in a pattern consistent with an inherited syndrome. Up to 25% of all cases of CRC may fall into this category (Paula and Harold, 2002).

### Classification and grading

The most common colon cancer cell type is adenocarcinoma which accounts for 95% of cases. Other, rarer types include lymphoma and squamous cell carcinoma. Cancers on the right side (ascending colon and cecum) tend to be exophytic, that is, the tumor grows outward from one location in the bowel wall. Left-sided tumors tend to be circumferential, and can obstruct the bowel much like a napkin ring.

Two classification systems are being used for the staging of the CRC-Dukes classification and TNM (Tumors/Nodes/Metastases) system. Dukes’ classification, first proposed by Dukes (1932), identifies the stages as: (A) Tumor confined to the intestinal wall; (B) Tumor invading through the intestinal wall; (C) With lymph node(s) involvement; and (D) With distant metastasis, which is the commonest in use still (Table 1). There has been a gradual move from using Dukes’s classification to using the TNM classification system as this is thought to lead to a more accurate, independent description of the primary tumors and its spread (Hardy et al., 2001).

**Table 1:** **Staging and survival of colorectal cancers**.

Duke’s staging	TNM staging	Description	Survival (%)
	Stage 0	Carcinoma *in situ*
A	Stage I	No nodal involvement, no metastases, tumor invades submucosa (T_1_, N_o_, M_o_); tumor invades muscularis propria (T_2_, N_o_, M_o_)	90–100
B	Stage II	No nodal involvement, no metastases, tumor invades subserosa (T_3_, N_o_, M_o_); tumor invades other organs (T_4_, N_o_, M_o_)	75–85
C	Stage III	Regional lymph nodes involved (any T, N_1_, M_o_)	30–40
D	Stage IV	Distant metastases	< 5

### Risk factors

A number of risk factors have been identified which increase the person’s risk of developing CRC. The most important of these are given in a Box 1, as has been identified by Mayo foundation.

Box 1 **Various risk factors affecting the predisposition to colorectal cancer**

Mayo Foundation for Medical Education and Research has indentified some of the factors the may increase the risk of colorectal cancer which are summarized below:

**Age:** Although there is no defined age bar at which colorectal cancer can occur but persons older than 50 are at a higher risk of developing colorectal cancer than younger people.

**Gender:** In developed countries, both men and women are affected equally while as in developing countries the men have been found to be affected more than their women counterparts.

**Ethnic Background/Race:** African-Americans have a greater risk of colorectal cancer than do people of other races. Jews of Eastern European descent (Ashkenazi Jews) may have a higher rate of colon cancer.

**Inflammatory intestinal conditions:** A person who has a history of Chrohn’s Disease and Inflammatory Bowel Disease has an increased chance of developing colorectal cancer.

**A Family history or personal of colorectal cancer or colon polyps:** A person is more likely to develop colorectal cancer if anyone of his/her parents; siblings or child is having the disease. If more than one family member has colon cancer or rectal cancer, the risk is even greater. So is the case with person who has had colon cancer or adenomatous polyps.

**Low-fiber, high-fat diet:** A diet made up mostly of foods that are high in fat, especially from animal sources, can increase the risk of colorectal cancer. Colorectal cancer may be associated with a diet low in fiber and high in calories also. Some studies have found an increased risk of colorectal cancer in people who eat diets high in red meat and processed meats.

**A sedentary lifestyle:** People who are not active have a higher risk of colorectal cancer. Smoking/Alcohol: Recent studies show that smokers are 30% to 40% more likely than nonsmokers to die of colorectal cancer. Heavy use of alcohol has also been linked to colorectal cancer.

**Diabetes:** People with diabetes and insulin resistance may have an increased risk of colon cancer.

**Obesity:** People who are obese have an increased risk of colon cancer and an increased risk of dying of colorectal cancer when compared with people considered normal weight.

**Inherited syndromes that increase colon cancer risk:** A person who has a specific inherited gene syndrome (such as Familial Adenomatous Polyposis (FAP) or Hereditary Non-Polyposis Colon Cancer (HNPCC) is at increased risk for developing colorectal cancer. Genetic syndromes passed through generations of the family can increase the risk of colorectal cancer.

**Radiation therapy for cancer:** Radiation therapy directed at the abdomen to treat previous cancers may increase the risk of colorectal cancer.

Box 2 **Symptoms in patients suffering from colorectal cancer**

The following symptoms may indicate colorectal cancer

- A change in bowel habits
- Diarrhea or constipation
- Feeling that the bowel does not empty completely
- Vomiting
- Blood in the stool
- Abdominal discomfort (gas, bloating, cramps)
- Weight loss of no known reasons
- Constant tiredness
- Unexplained anemia

Box 3 **Diagnostic tests recommended for colorectal cancer detection**

Approved screening and diagnostic tools include:

- Fecal Occult Blood Test (FOBT)
- Flexible Sigmoidoscopy
- Double Contrast Barium Enema (DCBE)
- Colonoscopy
- DNA-based Stool Test and Virtual Colonoscopy

## Genetic Background

The majority of CRCs develop from benign pre-neoplastic lesions: the adenomatous polyps or adenomas. A multistep model of carcinogenesis for the development of colorectal cancer have been given by Vogelstein which describes the progression if a benign adenoma to a malignant carcinoma through a series of well-defined histological stages (Vogelstein et al., 1988), this is known as the adenoma-carcinoma sequence model. This model describes an accumulation of genetic events, each conferring a selective growth advantage to an affected colorectal cell. These genetic changes ultimately result in uninhibited cell growth, proliferation, and clonal tumor development. The additive and cumulative effect of these somatic mutations has been shown to be the cause of sporadic colorectal cancer.

The salient features of the Vogelstein’s model of CRC carcinogenesis for sporadic cancers, can be conclusively drawn as: 1) the mutational activation of oncogenes and/or the inactivation of tumor suppressor genes results in colorectal carcinogenesis; 2) at least four or five genes of a cell must undergo somatic mutations so as to get malignantly transformed; 3) the characteristics of the tumor is dependent upon the accumulation of multiple genetic mutations rather than the sequence of mutations of the genes involved; and 4) the features of the tumorigenic process of colorectal cancer also apply to other solid tumors, such as breast, stomach and pancreatic cancer.

Two major mechanisms of genomic instability have been identified that give rise to colorectal carcinoma development and progression: chromosomal instability (CIN) and microsatellite instability (MIN).

### Chromosomal instability

It is associated with the series of genetic changes that occur initially in some cases by loss of one allele at a chromosomal locus (loss of heterozygosity) and may imply the presence of a tumor-suppressor gene at that site. Loss of both alleles at a given locus (homozygous deletion) is an even stronger indicator of the existence of a tumor-suppressor gene. Loss of heterozygosity occurs clonally in both the adenoma-carcinoma sequence and ulcerative colitis associated neoplasia. Many of these loci are already associated with one or more known candidate tumor-suppressor genes. These include 3p21 (β*-Catenin* gene), 5q21 (*APC* gene), 9p (*p16* and *p15* genes), 13q (retinoblastoma gene), 17p (*p53* gene), 17q (*BRCA1* gene), 18q (*DCC* and *SMAD4* genes), and less frequently 16q (*E-cadherin* gene) (Esteller et al., 2001; Conlin et al., 2005; Hsieh et al., 2005). FAP represents the hereditary syndrome dealing with *APC* gene mutation (Fearon and Vogelstein, 1990).

### Microsatellite instability

It comprises length alterations of oligonucleotide repeat sequences that occur somatically in human tumors. Mutations in DNA mismatch repair (MMR) genes result in a failure to repair errors that occur during DNA replication in repetitive sequences [microsatellites (MSI)], resulting in an accumulation of frameshift mutations in genes that contain MSI. This failure leads to MIN type of tumor and is the hallmark of HNPCC (Boland et al., 1998). MIN is also found in 12–15% of sporadic CRCs. MIN tumors are more frequently right-sided and poorly differentiated, and more often display unusual histological type (mucinous), and marked peri-tumoral and intra-tumoral lymphocytic infiltration (Dolcetti et al., 1999; Benatti et al., 2005). Microsatellite instability also occurs in patients with ulcerative colitis and is fairly common in premalignant (dysplastic) and malignant lesions (21 and 19%, respectively) (Kerr et al., 2001).

According to the Vogelstein’s model of CRC carcinogenesis the etiology of CRC is multifactorial, and is likely to involve the actions of genes at multiple levels of the carcinogenesis trail. Various genes which have been implicated in the pathogenesis of CRC include *p53*, *p16*, *p14*, *APC*, β*-catenin*, *E-cadherin*, *Transforming Growth Factor* (*TGF*)*-*β, *SMADs*, *MLH1*, *MSH2*, *MSH6*, *PMS2*, *AXIN*, S*TK11*, *PTEN*, *DCC*, and *KRAS* (Sayar and Banerjee, 2007).

### Molecular Mutations

The mutations whether point or gross mutations in a number of tumor suppressor or oncogenes have been implicated in the development of colorectal cancer in every corner and every population of the world. A number of important genes whose mutations have been ascertained to be present in the colorectal cancer have been discussed in brief as below.

#### TP53

The *TP53* gene is located on the short (p) arm of chromosome 17, and 17p deletions are found in 6–25% of colonic adenomas and in as many as 75% of colonic carcinomas (Baker et al., 1989). The *TP53* gene encodes a protein, which maintains genomic integrity by inducing cell cycle arrest and apoptosis when DNA is damaged (Levine et al., 1991). Mutations in *TP53* gene occur in almost half of all CRCs, proposed as a late event in the transition of an adenoma to carcinoma (Harris and Hollstein, 1993). The mutations in *p53* are thought to cause an increase in the half life of the protein and also often associated with overexpression in the nucleus, in one view (Remvikos et al., 1990). Also, most of the mutations in *TP53* gene occur in exons 5–8, in highly preserved regions, and in the three main structural domains of the *p53* protein (L2, L3, and loop-sheet-helix) (Borrensen-Dale et al., 1998). These mutations cause the synthesis of a stable protein that loses the ability to bind DNA and to cause the activation of target genes (Soussi and Beroud, 2001).

#### KRAS

*KRAS* gene located at 12p12 consists of six exons, spread over 35 kb of genomic DNA, alternative RNA splicing reveals two different transcripts of 5.5 and 3.8 kb. Ras proteins small monomeric proteins of 189 amino acids with a molecular weight of 21 Kd (Watzinger and Lion, 1999). These function as small GTPases that cycle between inactive guanosine diphosphate (GDP)-bound and active guanosine triphosphate (GTP)-bound conformations (Ras–GDP and Ras–GTP, respectively) (Boguski and McCormick, 1993; Donovan et al., 2002). The human ras family consists of three proto-oncogenes, *Harvey (H)-*, *Kirsten (K)-*, and *N-ras*, all of which possess an intrinsic GTPase activity, implicated in the regulation of their activity. RAS proteins control multiple pathways in a tissue-specific manner, affecting cell growth, differentiation, and apoptosis (Khosrave-far and Der, 1994). Specific mutations in the *RAS* genes lead to the formation of constitutively active proteins, which trigger the transduction of proliferative and/or differentiative signals, even in the absence of extracellular stimuli (Fearon and Vogelstein, 1990; Schubbert et al., 2007).

Human cancers frequently express mutant Ras proteins, termed “oncogenic Ras.” Activating *ras* mutations can be found in human malignancies with an overall frequency of 15–20% (Schubbert et al., 2007). Activating mutations in the *KRAS* proto-oncogene gene are involved in 25–60% of CRCs (Vogelstein et al., 1988; Fearon and Vogelstein, 1990). The activating oncogenic mutations, in particular of *KRAS* are found mostly (90%) in codons 12 and 13, but may also affect codon 61 (Fearon, 1994; Bazan et al., 2002). The most frequently observed are the G > A, G > T, and G > C transversions (Brink et al., 2005; Schubbert et al., 2007).

#### BRAF

*BRAF* gene is located at 7q34 in between NDUFB2 and MRPS33 genes. It is composed of 18 exons spanning in a region of 190 Kbp. Its mRNA has 2478 bp. *Braf* protein consists of 766 amino acid residues having MW of 85 Kd. The *BRAF* gene is a proto-oncogene that belongs to the Serine/Threonine Kinase Family. It is also a member of the *RAF* Subfamily together with the *ARAF* and *RAF1* genes. *Braf* protein contains three highly conserved domains RBD (Ras binding domain), CRD (Cysteine-rich domain), and KD (Kinase domain). Within the kinase domain there lies two other specific domains – one glycine motif (G-loop) in exon 11 and other activation segment (AS) in exon 15 (Domingo and Schwartz, 2004).

*BRAF* presents somatic mutations in different sort of tumors, predominantly in malignant melanoma, sporadic colorectal tumors showing MMR defects in MSI, low-grade ovarian serous carcinoma and thyroid papillary cancer. About 80% of these mutations correspond to the hotspot transversion mutation T1799A that causes the amino acidic substitution V600E (Davies et al., 2002) The other 20% accounts for a wide variable range of missense mutations and all of them reside in the glycines of the G-loop in the exon 11 or in the AS in exon 15 near the V600. The mutation V600E confers transformant activity to the cells because it mimics the phosphorylation of T599 and/or S602 in the AS and so Braf rests constitutively active in a RAS independent manner (Wan et al., 2004).

#### APC

*APC* is a classical tumor-suppressor gene located on 5q21 containing 21 exons. *APC* transcript is 9.0 kb in length and the most common isoform of Apc protein contains 2843 amino acids with molecular weight of 310 kD. The Apc protein consists of an oligomerization domain, armadillo region in the N-terminus, a number of 15- and 20-amino acid repeats in the central portion, and a C-terminus that contains a basic domain and binding sites for EB1 and the human disk large (HDLG) protein. Although being an integral part of the Wnt-signaling mechanism, it also plays a role in cell–cell adhesion, stability of microtubular cytoskeleton, cell cycle regulation, and possibly apoptosis (Fearnhead et al., 2001).

The *APC* gene product indirectly regulates transcription of a number of critical cell proliferation genes, through its interaction with the transcription factor β-catenin. Apc binding to β-catenin leads to ubiquitin-mediated beta catenin destruction; loss of Apc function increases transcription of beta catenin targets. These targets include cyclin D, C-myc, ephrins, and caspases. Apc also interacts with numerous actin and microtubule associated proteins. Apc itself stabilizes microtubules. Homozygous Apc truncation has been shown to affect chromosome attachment in cultured cells. Roles for Apc in cell migration have been demonstrated *in vitro* and in mouse models (Hamelin, 1998; Polakis, 2000; Tirnauer, 2005).

In addition to the mutational inactivation, hypermethylation of the gene promoter is the other important mechanism associated with gene silencing (Chen et al., 2005). In many tumors the hypermethylation of CpG islands in gene promoters has been found to be a frequent epigenetic change in cancers, and is usually associated with the loss of transcription of APC (Esteller et al., 2000; Rowan et al., 2000; Tsuchiya et al., 2000; Virmani et al., 2001; Esteller and Herman, 2002; Kang et al., 2003; Lind et al., 2004; Zare et al., 2009). Hypermethylation of the APC gene promoters has been reported to be present in about 20–48 per cent of human CRCs (Hiltunen et al., 1997; Esteller et al., 2000; Arnold et al., 2004; Lind et al., 2004).

#### β-Catenin

β*-Catenin* is located at 3p22-p21.3 and encompasses 23.2 kb of DNA containing 16 exons with mRNA transcript about 2343 bp long coding 781 amino acid residue protein of 92 kD molecular weight. β*-Catenin* protein contains a phosphorylation site by the serine-threonine glycogen synthase kinase-3β (GSK-3β), an α*-Catenin* binding site, 13 armadillo repeats, and a transactivating domain (from N-terminus to C-terminus). β*-Catenin* is assumed to transactivate mostly unknown target genes, which may stimulate cell proliferation (acts as an oncogene) or inhibit apoptosis. The β*-Catenin* level in the cell is regulated by its association with the adenomatous polyposis coli (*APC*) tumor-suppressor protein, axin, and *GSK-3*β. Phosphorylation of b-catenin by the APC-axin-GSK-3β complex leads to its degradation by the ubiquitin-proteasome system (Debuire et al., 2002).

β-*Catenin* is mutated in up to 10% of all sporadic colorectal carcinoma by point mutations or in frame deletions of the serine and threonine residues that are phosphorylated by GSK-3β (Polakis, 2000). These mutations result in stabilization of β*-Catenin* and activation of *WNT*-signaling. Mutations in β*-Catenin* occur in exclusivity to *APC* aberrations as both molecules are the components of the same pathway (Behrens, 2005).

#### SMAD4

*SMAD4* gene – also known as *MADH4*, *DPC4* & *JIP*, is located on the long arm (q) of chromosome 18 at band 21.1. The gene encompasses 49.5 kb of DNA with 13 exons, out of which first two exons do not code for any amino acid and hence constitute 5′-UTR of the *SMAD4* gene. *SMAD4* mRNA transcript constitutes 3220 nucleotides (Saffroy et al., 2004). The protein of *SMAD4* gene – *Smad4* belongs to the Darfwin family of proteins which harbors two conserved amino- and carboxyl-terminal domains known as MH1 and MH2, respectively. Smad4 in the basal state is found mostly as a homo-oligomer, most likely a trimer. It is ubiquitously expressed within the human body. Smad4 is an intracellular mediator of TGF-β family and activin type 1 receptor. Smad4 mediate TGF-β signaling to regulate cell growth and differentiation. TGF-β stimulation leads to phosphorylation and activation of Smad2 and Smad3, which form complexes with Smad4 that accumulate in the nucleus and regulate transcription of target genes. By interacting with DNA-binding proteins, Smad complexes then positively or negatively regulate the transcription of target genes (Attisano and Wrana, 2000; Massagué et al., 2000; Wrana, 2000; Attisano and Lee-Hoeflich, 2001; Shi, 2001).

The role of Smad4 gene as an important tumor-suppressor gene came out by the novel study of the allelotype loss in pancreatic adenocarcinoma (Shi, 2001). The tissue restriction of alterations in *DPC4*, as in many other tumor-suppressor genes, emphasizes the complexity of rate-limiting checkpoints in human tumorigenesis (Schutte et al., 1996).

Smad4 was proposed to be a tumor-suppressor gene that may function to disrupt TGF-β signaling. Mutant Smad4 proteins, identified in human carcinomas, were found to be impaired in their ability to regulate gene transcription. Most of Smad4 gene mutations in human cancer are missense, nonsense, and frameshift mutations at the mad homology 2 region (MH2) which interfere with the homo-oligomer formation of Smad4 protein and hetero-oligomer formation between Smad4 and Smad2 proteins, resulting in disruption of TGF-β signaling (Shi, 2001; Woodford-Richens et al., 2001; Roth et al., 2003).

#### AXIN

Axis inhibition protein (*AXIN1*) and its homolog *AXIN2* (also known as conductin) are tumor-suppressor genes and their proteins act as master scaffold ones. *AXIN1* is located on 16p13.3 while as *AXIN2* is located 17q24.1 (Atlas.org). Axin has plieotrophic effect on various signaling pathways. One of its key functions is to negatively regulate the activity of the *WNT* pathway by enhancing formation of the β-Catenin destruction complex. The Wnt/Wingless biological signaling pathway plays an important role in both embryonic development and tumorigenesis (Hong-Tao et al., 2007). The genomic region containing the *AXIN1/2* genes shows loss of heterozygosity and re-arrangements in a variety of cancers. In addition somatic point mutations and deletions have been identified in CRC, hepatocellular carcinomas, ovarian endometrioid adenocarcinomas, and hepatoblastomas. Many of these mutations/deletions result in translation of truncated proteins that are likely to be functionally inactive (Lammi et al., 2004).

### Single-nucleotide polymorphisms

The human genome contains a massive amount of genetic variation, such as the insertion/deletion of one or more nucleotides, the copy-number variations (CNVs) that can involve DNA sequences of a few kilobases up to millions of bases, and single-nucleotide polymorphisms (SNPs), which are the substitution of a single-nucleotide along the DNA (Ionita-Laza et al., 2009; Savas and Liu, 2009). SNPs are the most common form of genetic variation. There are >10 million SNPs estimated to be in the human genome (Miller et al., 2005).

These days several molecular and epidemiological studies are focusing on the role of SNP’s in modulating the risk of various cancer and quite a number of studies have implicated various gene polymorphisms in affecting the risk of cancer in almost all the populations around the world.

#### TP53 polymorphism

In *TP53* gene several polymorphisms have been identified both in non-coding and coding regions (Murphy, 2006; Bojesen and Nordestgaard, 2008; Costa et al., 2008; Whibley et al., 2009). Most of these polymorphisms are SNPs affecting a single base. Within the coding regions of TP53, only two important polymorphisms are present which alter the amino acid sequence of their products (Pietsch et al., 2006), these are located at codon 47 and codon 72 in exon 4. Codon 72 (Arg72Pro) – a frequent functional SNP that leads to an arginine-proline amino acid change has been reported by many authors (Thomas et al., 1999; Dumont et al., 2003). Dumont et al., reported that the Arg72 allele, if in homozygous, has an apoptosis-inducing ability 15-fold higher than does the Pro72 allele. According to Leu et al. (2004), this high apoptosis-inducing ability of the Arg72 allele is in part due to its mitochondrial location which makes it possible for TP53 to have a direct interaction with pro-apoptotic BAK protein. Studies on this SNP function were the basis for testing its impact on the risk and progression of tumors, where the less apoptotic allele Pro72 was associated with increased risk for development of tumors (Marin et al., 2000; Ignaszak-Szczepaniak et al., 2006; Toyama et al., 2007). Codon 47 (Pro47Ser) – second most common polymorphism in TP53 that leads to change of Proline with Serine was first identified by Felley-Bosco et al. (1993). The Ser47 polymorphic variant is very rare, with an allele frequency under 5% in populations of African origin (Murphy, 2006; Pietsch et al., 2006; Whibley et al., 2009). In a pioneer study by Li et al. (2005), 106, it was found that the serine 47 polymorphic variant, which replaces the proline residue – necessary for recognition by proline-directed kinases, is a markedly poorer substrate for phosphorylation. Codon 47 encodes proline (CCG) in wild-type p53, but in a small subset of individuals it can encode serine (TCG).

#### NQO1 polymorphism

*NQO1* is located on chromosome 16q22, is 20 kb in length and has 6 exons and 5 introns. *NQO1* is a flavoprotein which functions as a homodimer. The physiological dimer has one catalytic site per monomer. Each monomer consists of 273 amino acids. *NQO1* is expressed in human epithelial and endothelial tissues and at high levels throughout many human solid tumors. *NQO1* is a mainly cytosolic enzyme although it has also been localized in smaller amounts to mitochondria, endoplasmic reticulum and nucleus (Ross, 2004; Chao et al., 2006). The enzyme is generally considered as a detoxification enzyme because of its ability to reduce reactive quinones and quinone-imines to less reactive and less toxic hydroquinones by its unique ability to use either NADH or NADPH as reducing cofactors (Siegel et al., 2004). Because of its reducing capability *NQO1* prevents the generation of semiquinone free radicals and reactive oxygen species with its unique property of transferring two electrons at a time to quinone, thus protecting cells from oxidative damage (Chen et al., 1999; Winski et al., 2002).

#### CYP2E polymorphism

*Cytochrome P450 2E1* (*CYP2E1*) gene is located on 10q26.3. It is 18,754 bp long consisting of nine exons and eight introns, which encodes a 493 amino acid protein. *CYP2E1* belongs to the cytochrome P450 superfamily (Wang et al., 2010). It is a natural ethanol-inducible enzyme that is of great interest due to its role in the metabolism and bioactivation of many low molecular weight compounds, including ethanol, acetone, drugs like acetaminophen, isoniazid, chlorzoxazone, and fluorinated anesthetics and many procarcinogens like benzene, N-nitrosoamines, vinyl chloride, and styrene (Guengerich et al., 1991; Kharasch and Thummel, 1993; Ulusoy et al., 2007; Zhou et al., 2010). *CYP2E1* gene contains six restriction fragment length polymorphisms, of these are the two important – *Rsa*I polymorphism (CYP2E1*5B; C-1054T substitution) and the 96-bp insertion in its 5′-flanking region have drawn much interest (Morita et al., 2008; Wang et al., 2010; Zhou et al., 2010). *Rsa*I polymorphism has been shown to affect its transcription level. The variant type of this polymorphic site can enhance the transcription and increase the level of *CYP2E1* enzymatic activity *in vitro* (Hayashi et al., 1991). The variant allele of the 96-bp insertion polymorphism was shown to express greater transcriptional activity (Nomura et al., 2003).

#### MTHFR polymorphism

The *MTHFR* gene, located on 1p36.22, encompasses 19.3 kb of DNA and is composed of 11 exons. The gene codes for a 74.6-kD protein of 656 amino acids (Saffroy et al., 2005). It is a cytosolic enzyme that catalyzes the conversion of 5,10-methylene tetrahydrofolate (THF) to 5-methylTHF, a cosubstrate for homocysteine remethylation to methionine with subsequent production of S-adenosyl methionine (SAM), the universal methyl donor in humans, required for DNA methylation. The methylation of homocysteine is catalyzed by the enzyme methionine synthase, which requires the cofactor vitamin B12. *MTHFR* is also linked to the production of dTMP via thymidylate synthase and to purine synthesis and, therefore, plays a role in the provision of nucleotides essential for DNA synthesis (Wagner, 1995). Thus, any defect in the *MTHFR* gene will be reflected in a defect in the methylation pattern of DNA as well as in its synthesis. Two common functional polymorphisms have been defined in the *MTHFR* gene – one is *C677T* and other *A1298C*. *MTHFR C677T* polymorphism is the most important one regulating the function of this enzyme. This polymorphism results in an alanine-to-valine substitution at codon 222 of the protein (Frosst et al., 1995). This polymorphism has a profound effect on the MTHFR protein, not only does it decrease the thermal stability of this enzyme but also reduces its activity (Cicek et al., 2004). Individuals with the variant Val/Val genotype (TT) have no more than 30% of normal enzyme activity, and heterozygotes (CT) have 65% of normal enzyme activity (Frosst et al., 1995; Kono and Chen, 2005). This substitution also results in lower levels of 5-methyltetrahydrofolate, an accumulation of 5,10-methylenetetrahydrofolate and increased plasma homocysteine levels (Frosst et al., 1995; Ma et al., 1997; Bagley and Selhub, 1998).

## Conclusion

However, there is an unresolved debate among the geneticists and molecular oncologists as to whether which of the two pathways – CIN and MIN initiates and predisposes an individual to the development of colorectal cancer. Still it is not clear which of the two is the first event in the carcinogenesis. However, as *per se* it is regarded that CIN is the earlier event in the tumorigenesis and subsequently tumor progression. In addition there is the question of the epigenetic silencing of the various CIN and MIN pathway genes also which occurs exclusive or in addition to the mutations of the genes thus adding a second dimension of complexity in the molecular mechanism of the tumor development.

Also molecular biologist are fighting to double their efforts to define and characterize various metabolic pathways associated with DNA structure and function like DNA methylation and chromatin modification, changes in the patterns of mRNA and noncoding RNA expression, and the consequent effect on the corresponding protein expression and posttranslational modification which surely are the variables affecting the CRC tumorigenesis. So, there are more than one pathway for the tumor to undergo tumorigenesis and hence it is important for the molecular oncologist to obtain data about age, sex, tumor site, ethinicity, diet and gut flora when investigating genetic and epigenetic risk factors for CRC to understand the complex interactions among dietary and environmental agents.

## Conflict of Interest Statement

The authors declare that the research was conducted in the absence of any commercial or financial relationships that could be construed as a potential conflict of interest.

## Footnotes

^1^http://www.cancer.gov/cancertopics/pdq/genetics/colorectal/healthprofessional/allpages

## References

[B1] Arnold C. N. Goel A. Niedzwiecki D. Dowell J. M. (2004). APC promoter hypermethylation contributes to the loss of APC expression in colorectal cancers with allelic loss on 5q. Cancer Biol. Ther. 3, 960–964 10.4161/cbt.3.10.1113 15326380

[B2] Attisano L. Lee-Hoeflich S. T. (2001). The Smads. Genome Biol. 2, 3010.1–3010.8 10.1186/gb-2001-2-8-reviews3010 11532220 PMC138956

[B3] Attisano L. Wrana J. L. (2000). Smads as transcriptional co-modulators. Curr. Opin. Cell Biol. 12, 235–243 10.1016/S0955-0674(99)00081-2 10712925

[B4] Bagley P. J. Selhub J. A. (1998). common mutation in the methylenetetrahydrofolate reductase gene is associated with an accumulation of formylated tetrahydrofolates in red blood cells. Proc. Natl. Acad. Sci. U.S.A. 95, 13217–13220 10.1073/pnas.95.22.13217 9789068 PMC23763

[B5] Baker S. K. Fearon E. R. Nigro J. M. Hamilton S. R. Preisinger A. C. Jessup J. M. (1989). Chromosome 17 deletions and p53 gene mutations in colorectal carcinoma. Science 244, 217–221 10.1126/science.2649981 2649981

[B6] Bazan V. Migliavacca M. Zanna I. Tubiolo C. Grassi N. Latteri M. A. (2002). Specific codon 13 K-ras mutations are predictive of clinical outcome in colorectal cancer patients, whereas codon 12 K-ras mutations are associated with mucinous histotype. Ann. Oncol. 13, 1438–1446 10.1093/annonc/mdf226 12196370

[B7] Behrens J. (2005). The role of the Wnt signalling pathway in colorectal tumorigenesis. Biochem. Soc. Trans. 33, 672–676 10.1042/BST0330672 16042571

[B8] Benatti P. Gafa R. Barana D. Marino M. Scarselli A. Pedroni M. (2005). Microsatellite instability and CRC prognosis. Clin. Cancer Res. 11, 8332–8340 10.1158/1078-0432.CCR-05-1030 16322293

[B9] Boguski M. McCormick F. (1993). Proteins regulating Ras and its relatives. Nature 366, 643–653 10.1038/366643a0 8259209

[B10] Bojesen S. E. Nordestgaard B. G. (2008). The common germline Arg72Pro polymorphism of p53 and increased longevity in humans. Cell Cycle 7, 158–163 10.4161/cc.7.2.5249 18256523

[B11] Boland C. R. Thibodeau S. N. Hamilton S. R. Sidransky D. Eshleman J. R. Burt R. W. (1998). National Cancer Institute workshop on microsatellite instability for cancer detection and familial predisposition: development of international criteria for the determination of microsatellite instability in CRC. Cancer Res. 58, 5248–5257 9823339

[B12] Borrensen-Dale A. Lothe R. A. Meling G. I. Hainut P. Rognum T. O. Skovlund E. (1998). TP53 and long-term prognosis in colorectal cancer: mutations in the L3 zinc-binding domain predict poor survival. Clin. Cancer Res. 4, 203–210 9516972

[B13] Brink M. Weijenberg M. P. de Goeij A. F. Roemen G. M. Lentjes M. H. de Bruine A. P. (2005). Meat consumption and K-ras mutations in sporadic colon and rectal cancer in The Netherlands cohort study. Br. J. Cancer 92, 1310–1320 10.1038/sj.bjc.6602491 15812479 PMC2361976

[B14] Center M. M. Jemal A. Smith R. A. Ward E. (2009). Worldwide variations in colorectal cancer. CA Cancer J. Clin. 59, 366–378 10.3322/caac.20038 19897840

[B15] Chao C. Zhang Z.-F. Berthiller J. Boffetta P. Hashibe M. N. A. D. (2006). (P)H:quinone oxidoreductase 1 (NQO1) Pro187Ser polymorphism and the risk of lung. Bladder, and colorectal cancers: a meta-analysis. Cancer Epidemiol. Biomarkers Prev. 15, 979–987 10.1158/1055-9965.EPI-05-0899 16702380

[B16] Chen H. Lum A. Seifried A. Wilkens L. R. Marchand L. L. (1999). Association of the NAD(P)H:quinone oxidoreductase 609C3T polymorphism with a decreased lung cancer risk1. Cancer Res. 59, 3045–3048 10397241

[B17] Chen J. Röcken C. Lofton-Day C. Schulz H. U. Müller O. Kutzner N. (2005). Molecular analysis of APC promoter methylation and protein expression in colorectal cancer metastasis. Carcinogenesis 26, 37–43 10.1093/carcin/bgi052 15375009

[B18] Cicek M. S. Nock N. L. Li L. Conti D. V. Casey G. Witte J. S. (2004). Relationship between methylenetetrahydrofolate reductase C677T and A1298C genotypes and haplotypes and prostate cancer risk and aggressiveness. Cancer Epidemiol. Biomarkers Prev. 13, 1331–1336 15298954

[B19] Conlin A. Smith G. Carey F. A. Wolf C. R. Steele R. J. (2005). The prognostic significance of K-ras, p53, and APC mutations in colorectal carcinoma. Gut 54, 1283–1286 10.1136/gut.2005.066514 15843421 PMC1774675

[B20] Costa S. Pinto D. Pereira D. Rodrigues H. Cameselle-Teijeiro J. Medeiros R. (2008). Importance of TP53 codon 72 and intron 3 duplication 16bp polymorphisms in prediction of susceptibility on breast cancer. BMC Cancer 8:32 10.1186/1471-2407-8-32 18230179 PMC2254432

[B21] Davies H. Bignell G. R. Cox C. Stephens P. Edkins S. Clegg S. (2002). Mutations of the BRAF gene in human cancer. Nature 417, 949–954 10.1038/nature00766 12068308

[B22] Debuire B. Lemoine A. Saffroy R. (2002). CTNNB1 (catenin, beta-1). Atlas Genet. Cytogenet. Oncol. Haematol. Available at: http://AtlasGeneticsOncology.org/Genes/CTNNB1ID71.html

[B23] Dolcetti R. Viel A. Doglioni C. Russo A. Guidoboni M. Capozzi E. (1999). High prevalence of activated intraepithelial cytotoxic T lymphocytes and increased neoplastic cell apoptosis in colorectal carcinomas with microsatellite instability. Am. J. Pathol. 154, 1805–1813 10.1016/S0002-9440(10)65436-3 10362805 PMC1866613

[B24] Domingo E. Schwartz S. Jr. (2004). BRAF. Atlas Genet. Cytogenet. Oncol. Haematol. Available at: http://AtlasGeneticsOncology.org/Genes/BRAFID828.html

[B25] Donovan S. Shannon K. M. Bollag G. (2002). GTPase activating poteins: critical regulators of intracellular signaling. BBA Rev. Cancer 1602, 23–45 10.1016/s0304-419x(01)00041-5 11960693

[B26] Dukes C. E. (1932). The classification of cancer of the rectum. J. Pathol. Bacteriol. 35, 323 10.1002/path.1700350303

[B27] Dumont P. Leu J. I. Della Pietra A. C. III George D. L. Murphy M. (2003). The codon 72 polymorphic variants of p53 have markedly different apoptotic potential. Nat. Genet. 33, 357–365 10.1038/ng1093 12567188

[B28] Esteller M. Gonzalez S. Risques R. A. Marcuello E. Mangues R. Germa J. R. (2001). RAS and p16 aberrations confer poor prognosis in human CRC. J. Clin. Oncol. 19, 299–304 11208819 10.1200/JCO.2001.19.2.299

[B29] Esteller M. Herman J. G. (2002). Cancer as an epigenetic disease: DNA methylation and chromatin alterations in human tumours. J. Pathol. 196, 1–7 10.1002/path.1024 11748635

[B30] Esteller M. Sparks A. Toyota M. Sanchez-Cespedes M. Capella G. Peinado M. A. (2000). Analysis of adenomatous polyposis coli promoter hypermethylation in human cancer. Cancer Res. 60, 4366–4371 10969779

[B31] Fearnhead N. S. Britton M. P. Bodmer W. F. (2001). The ABC of APC. Hum. Mol. Genet. 10, 721–733 10.1093/hmg/10.7.721 11257105

[B32] Fearon E. R. (1994). Molecular genetic studies of the adenoma-carcinoma sequence. Adv. Intern. Med. 39, 123–147 8140953

[B33] Fearon E. R. Vogelstein B. (1990). A genetic model for colorectal tumorigenesis. Cell 61, 759–767 10.1016/0092-8674(90)90186-I 2188735

[B34] Felley-Bosco E. Weston A. Cawley H. M. Bennett W. P. Harris C. C. (1993). Functional studies of a germ-line polymorphism at codon 47 within the p53 gene. Am. J. Hum. Genet. 53, 752–759 8352280 PMC1682404

[B35] Frosst P. Blom H. J. Milos R. Goyette P. Sheppard C. A. Matthews R. G. (1995). A candidate genetic risk factor for vascular disease: a common mutation in methylenetetrahydrofolate reductase. Nat. Genet. 10, 111–113 10.1038/ng0595-111 7647779

[B36] Guengerich F. P. Kim D. H. Iwasaki M. (1991). Role of human cytochrome P450 IIE1 in the oxidation of many low molecular weight cancer suspects. Chem. Res. Toxicol. 4, 168–179 10.1021/tx00022a003 1664256

[B37] Hamelin P. (1998). APC (adenomatous polyposis coli). Atlas Genet. Cytogenet. Oncol. Haematol. Available at: http://AtlasGeneticsOncology.org/Genes/APC118.html

[B38] Hardy R. G. Meltzer S. T. Jankowski J. A. (2001). “Molecular basis for risk factors,” in ABC of CRC, eds Kerr D. J. Young A. N. Hobbs F. D. R. (London: British Medical Journal Books), 5

[B39] Harris C. C. Hollstein M. (1993). Clinical implication of the p53 tumor suppressor gene. N. Engl. J. Med. 329, 1318–1327 10.1056/NEJM199310283291807 8413413

[B40] Hayashi S. Watanabe J. Kawajiri K. (1991). Genetic polymorphisms in the 5’-flanking region change transcriptional regulation of the human cytochrome P450IIE1 gene. J. Biochem. 110, 559–565 1778977 10.1093/oxfordjournals.jbchem.a123619

[B41] Hiltunen M. O. Alhonen L. Koistinaho J. Myöhänen S. Kashiwaba M. Osakabe M. (1997). Hypermethylation of the APC (adenomatous polyposis coli) gene promoter region in human colorectal carcinoma. Int. J. Cancer 70, 644–648 10.1002/(SICI)1097-0215(19970317)70:6<644::AID-IJC3>3.0.CO;2-V 9096643

[B42] Hong-Tao X. Qiang W. Yang L. Lian-He Y. Shun-Dong D. Yang H. (2007). Overexpression of axin downregulates TCF-4 and inhibits the development of lung cancer. Ann. Surg. Oncol. 14, 3251–3259 10.1245/s10434-007-9555-9 17768662

[B43] Hsieh J. S. Lin S. R. Chang M. Y. Chen F. M. Lu C. Y. Huang T. J. (2005). APC. K-ras, and p53 gene mutations in CRC patients: correlation to clinicopathologic features and postoperative surveillance. Am. Surg. 71, 336–343 15943410

[B44] Ignaszak-Szczepaniak M. Horst-Sikorska W. Sawicka J. Kaczmarek M. Slomski R. (2006). The TP53 codon 72 polymorphism and predisposition to adrenocortical cancer in Polish patients. Oncol. Rep. 16, 65–71 16786124

[B45] Ionita-Laza I. Rogers A. J. Lange C. Raby B. A. Lee C. (2009). Genetic association analysis of copy-number variation (CNV) in human disease pathogenesis. Genomics 93, 22–26 10.1016/j.ygeno.2008.08.012 18822366 PMC2631358

[B46] Kang G. H. Lee S. Kim J. S. Jung H. Y. (2003). Profile of aberrant CpG island methylation along the multistep pathway of gastric carcinogenesis. Lab. Invest. 83, 635–641 12746473 10.1097/01.lab.0000067481.08984.3f

[B47] Kerr D. J. Young E. M. Hobbs F. D. R. (2001). ABC Of Colorectal Cancer. 1st Edn, London: British Medical Journal

[B48] Kharasch E. D. Thummel K. E. (1993). Identification of cytochrome P450 2E1 as the predominant enzyme catalyzing human liver microsomal defluorination of sevoflurane, isoflurane, and methoxyflurane. Anesthesiology 79, 795–807 10.1097/00000542-199310000-00023 8214760

[B49] Khosrave-far R. Der C. J. (1994). The ras signal transduction pathway. Cancer Metastasis Rev. 13, 67–89 10.1007/BF00690419 8143346

[B50] Kono S. Chen K. (2005). Genetic polymorphisms of methylenetetrahydrofolate reductase and colorectal cancer and adenoma. Cancer Sci. 96, 535–542 10.1111/j.1349-7006.2005.00090.x 16128738 PMC11160001

[B51] Lammi L. Arte S. Somer M. Jarvinen H. Lahermo P. Thesleff I. (2004). Mutations in AXIN2 cause familial tooth agenesis and predispose to colorectal cancer. Am. J. Hum. Genet. 74, 1043–1050 10.1086/386293 15042511 PMC1181967

[B52] Leu J. I. Dumont P. Hafey M. Murphy M. E. George D. L. (2004). Mitochondrial p53 activates Bak and causes disruption of a Bak-Mcl1 complex. Nat. Cell Biol. 6, 443–450 10.1038/ncb1123 15077116

[B53] Levine A. J. Momand J. Finlay C. A. (1991). The p53 tumour suppressor gene. Nature 351, 453–456 10.1038/351453a0 2046748

[B54] Li X. Dumont P. Della P. A. Shetler C. Murphy M. E. (2005). The codon 47 polymorphism in p53 is functionally significant. J. Biol. Chem. 280, 24245–24251 10.1074/jbc.M414139200 15851479

[B55] Lind G. E. Thorstensen L. Løvig T. Meling G. I. Hamelin R. Rognum T. O. (2004). A CpG island hypermethylation profile of primary colorectal carcinomas and colon cancer cell lines. Mol. Cancer 3, 28 10.1186/1476-4598-3-28 15476557 PMC526388

[B56] Ma J. Stampfer M. J. Giovannucci E. Artigas C. Hunter D. J. Fuchs C. (1997). Methylenetetrahydrofolate reductase polymorphism, dietary interactions, and risk of colorectal cancer. Cancer Res. 57, 1098–1102 9067278

[B57] Marin M. C. Jost C. A. Brooks L. A. Irwin M. S. O’Nions J. Tidy J. A. (2000). A common polymorphism acts as an intragenic modifier of mutant p53 behaviour. Nat. Genet. 25, 47–54 10.1038/75586 10802655

[B58] Massagué J. Blain S. W. Lo R. S. (2000). TGF-β signaling in growth control, cancer and heritable disorders. Cell 103, 295–309 10.1016/S0092-8674(00)00121-5 11057902

[B59] Miller R. D. Phillips M. S. Jo I. Donaldson M. A. Studebaker J. F. Addleman N. (2005). High-density single-nucleotide polymorphism maps of the human genome. Genomics 86, 117–126 10.1016/j.ygeno.2005.04.012 15961272 PMC1885222

[B60] Morita M. Tabata S. Tajima O. Yin G. Abe H. Kono S. (2008). Genetic polymorphisms of CYP2E1 and risk of colorectal adenomas in the self defense forces health study. Cancer Epidemiol. Biomarkers Prev. 17, 1800–1807 10.1158/1055-9965.EPI-08-0314 18628434

[B61] Murphy M. E. (2006). Polymorphic variants in the p53 pathway. Cell Death Differ. 13, 916–920 10.1038/sj.cdd.4401907 16557270

[B62] Nomura F. Itoga S. Uchimoto T. Tomonaga T. Nezu M. Shimada H. (2003). Transcriptional activity of the tandem repeat polymorphism in the 5′-flanking region of the human CYP2E1 gene. Alcohol. Clin. Exp. Res. 27, 42S–46S 10.1097/01.ALC.0000078612.01626.96 12960506

[B63] O’Brien M. J. Winawer S. J. Zauber A. G. (2004). Flat adenomas in the national polyp study: is there increased risk for high-grade dysplasia initially or during surveillance? Clin. Gastroenterol. Hepatol. 2, 905–911 10.1016/S1542-3565(04)00392-1 15476154

[B64] Paula M. C. Harold F. (2002). The genetics of CRC. Ann. Intern. Med. 137, 603–612 10.7326/0003-4819-137-7-200210010-00012 12353948

[B65] Pietsch E. C. Humbey O. Murphy M. E. (2006). Polymorphisms in the p53 pathway. Oncogene 25, 1602–1611 10.1038/sj.onc.1209367 16550160

[B66] Polakis P. (2000). Wnt signaling and cancer. Genes Dev. 14, 1837–1851 10921899

[B67] Remvikos Y. Laurent-Puig P. Salmon R. J. Frelat G. Dutrillaux B. Thomas G. (1990). Simultaneous monitoring of p53 protein and DNA content of colorectal adenocarcinomas by flow cytometry. Int. J. Cancer 45, 450–456 10.1002/ijc.2910450313 2137812

[B68] Ross D. (2004). NQO1. Atlas Genet. Cytogenet. Oncol. Haematol. Available at: http://AtlasGeneticsOncology.org/Genes/NQO1ID375.html

[B69] Roth S. Johansson M. Loukola A. Peltomaki P. Jarvinen H. Mecklin J.-P. (2003). Mutation analysis of SMAD2, SMAD3, and SMAD4 genes in hereditary non-polyposis CRC. J. Cell. Sci. 11, 413–419 10.1136/jmg.37.4.298 PMC1734557 10819637

[B70] Rowan A. J. Lamlum H. Ilyas M. Wheeler J. Straub J. Papadopoulou A. (2000). APC mutations in sporadic colorectal tumors: A mutational “hotspot” and interdependence of the “two hits.” Proc. Natl. Acad. Sci. U.S.A. 97, 3352–3357 10.1073/pnas.97.7.3352 10737795 PMC16243

[B71] Saffroy R. Lemoine A. Debuire B. (2005). MTHFR (5,10-Methylenetetrahydrofolate reductase). Atlas Genet Cytogenet Oncol Haematol. Available at: http://AtlasGeneticsOncology.org/Genes/MTHFRID41448ch1p36.html

[B72] Saffroy R. Lemoine A. Debuire B. (2004). SMAD4 (mothers against decapentaplegic homolog 4 (Drosophila)). Atlas Genet. Cytogenet. Oncol. Haematol. Available at: http://AtlasGeneticsOncology.org/Genes/SMAD4ID371.html

[B73] Savas S. Liu G. (2009). Studying genetic variations in cancer prognosis (and risk): a primer for clinicians. Oncologist 14, 657–666 10.1634/theoncologist.2009-0042 19581524

[B74] Sayar N. Banerjee S. (2007). Colon: Colorectal adenocarcinoma. Atlas Genet Cytogenet. Oncol. Haematol. Available at: http://AtlasGeneticsOncology.org/Tumors/colon5006.html

[B75] Schubbert S. Shannon K. Bollag G. (2007). Hyperactive Ras in developmental disorders and Cancer. Nat. Rev. Cancer 7, 295–308 10.1038/nrc2175 17384584

[B76] Schutte M. Hruban R. H. Hedrick L. Cho K. R. Nadasdy G. M. Weinstein C. L. (1996). DPC4 gene in various tumor types. Cancer Res. 56, 2527–2530 8653691

[B77] Shi Y. (2001). Structural insights on Smad function in TGF-β signaling. Bioessays 23, 223–232 10.1002/1521-1878(200103)23:3<223::AID-BIES1032>3.0.CO;2-U 11223879

[B78] Siegel D. Gustafson D. L. Dehn D. L. Han J. Y. Boonchoong P. Berliner L. J. (2004). NAD(P)H:quinone oxidoreductase 1: role as a superoxide scavenger. Mol. Pharmacol. 65, 1238–1247 10.1124/mol.65.5.1238 15102952

[B79] Soussi T. Beroud C. (2001). Assessing TP53 status in human tumours to evaluate clinical outcome. Nat. Rev. Cancer 1, 233–240 10.1038/35105088 11902578

[B80] Thomas M. Kalita A. Labrecque S. Pim D. Banks L. Matlashewski G. (1999). Two polymorphic variants of wild-type p53 differ biochemically and biologically. Mol. Cell. Biol. 19, 1092–1100 9891044 10.1128/mcb.19.2.1092 PMC116039

[B81] Tirnauer J. (2005). APC (adenomatous polyposis coli). Atlas Genet. Cytogenet. Oncol. Haematol. Available at: http://AtlasGeneticsOncology.org/Genes/APC118.html

[B82] Toyama T. Zhang Z. Nishio M. Hamaguchi M. Kondo N. Iwase H. (2007). Association of TP53 codon 72 polymorphism and the outcome of adjuvant therapy in breast cancer patients. Breast Cancer Res. 9, R34 10.1186/bcr1682 17537232 PMC1929098

[B83] Tsuchiya T. Tamura G. Sato K. Endoh Y. Sakata K. Jin Z. (2000). Distinct methylation patterns of two APC gene promoters in normal and cancerous gastric epithelia. Oncogene 19, 3642–3646 10.1038/sj.onc.1203704 10951570

[B84] Ulusoy G. Arinç E. Adali O. (2007). Genotype and allele frequencies of polymorphic CYP2E1 in the Turkish population. Arch. Toxicol. 81, 711–718 10.1007/s00204-007-0200-y 17380320

[B85] Vainio H. Miller A. B. (2003). Primary and secondary prevention in CRC. Acta Oncol. 42, 809–815 10.1080/02841860310010673 14968941

[B86] Virmani A. K. Rathi A. Sathyanarayana U. G. Padar A. Huang C. X. Cunnigham H. T. (2001). Aberrant methylation of the adenomatous polyposis coli (APC) gene promoter 1A in breast and lung carcinomas. Clin. Cancer Res. 7, 1998–2004 11448917

[B87] Vogelstein B. Fearon E. R. Hamilton S. R. Kern S. E. Preisinger A. C. Leppert M. (1988). Genetic alterations during colorectal-tumor development. N. Engl. J. Med. 319, 525–532 10.1056/NEJM198809013190901 2841597

[B88] Wagner C. (1995). “Biochemical role of folate in cellular metabolism,” in Folate in Health and Disease, ed. Bailey L. B. (New York: Marcel Dekker), 23–42

[B89] Wan P. T. Garnett M. J. Roe S. M. Lee S. Niculescu-Duvaz D. Good V. M. (2004). Mechanism of activation of the RAF-ERK signaling pathway by oncogenic mutations of B-RAF. Cell 116, 855–867 10.1016/S0092-8674(04)00215-6 15035987

[B90] Wang Y. Yang H. Li L. Wang H. Zhang C. Yin G. (2010). Association between CYP2E1 genetic polymorphisms and lung cancer risk: a meta-analysis. Eur. J. Cancer 46, 758–764 10.1016/j.ejca.2010.07.008 20031389

[B91] Watzinger F. Lion T. (1999). RAS family. Atlas Genet. Cytogenet. Oncol. Haematol. Available at: http://AtlasGeneticsOncology.org/Deep/Ras.html

[B92] Whibley C. Pharoah P. D. P. Hollstein M. (2009). p53 Polymorphisms: cancer implications. Nat. Rev. Cancer 9, 95–107 10.1038/nrc2584 19165225

[B93] Winski S. L. Koutalos Y. Bentley D. L. Ross D. (2002). Subcellular localization of NAD(P)H: quinone oxidoreductase 1 inhuman cancer cells. Cancer Res. 62, 1420–1424 11888914

[B94] Woodford-Richens K. L. Rowan A. J. Poulsom R. Bevan S. Salovaara R. Aaltonen L. A. (2001). Comprehensive analysis of SMAD4 mutations and protein expression in juvenile polyposis: evidence for a distinct genetic pathway and polyp morphology in SMAD4 mutation carriers. Am. J. Pathol. 159, 1293–1300 10.1016/S0002-9440(10)62516-3 11583957 PMC1850516

[B95] Wrana J. L. (2000). Regulation of Smad activity. Cell 100, 189–192 10.1016/S0092-8674(00)81556-1 10660041

[B96] Zare M. Jazii F. R. Alivand M. R. Nasseri N. K. Malekzadeh R. Yazdanbod M. (2009). Qualitative analysis of adenomatous polyposis coli promoter: Hypermethylation, engagement and effects on survival of patients with esophageal cancer in a high risk region of the world, a potential molecular marker. BMC Cancer 9:24 10.1186/1471-2407-9-24 19149902 PMC2637891

[B97] Zauber A. G. O’Brien M. J. Winawer S. J. (2002). On finding flat adenomas: is the search worth the gain? Gastroenterology 122, 839–840 10.1053/gast.2002.32125 11878297

[B98] Zhou G. W. Hu J. Li Q. (2010). CYP2E1 Pst/Rsa polymorphism and colorectal cancer risk: a meta-analysis. World J. Gastroenterol. 16, 2949–2953 10.3748/wjg.v16.i18.2291 20556843 PMC2887593

